# Construction of a lncRNA–mRNA Co-Expression Network for Nasopharyngeal Carcinoma

**DOI:** 10.3389/fonc.2022.809760

**Published:** 2022-07-07

**Authors:** Chunmei Fan, Fang Xiong, Yanyan Tang, Panchun Li, Kunjie Zhu, Yongzhen Mo, Yumin Wang, Shanshan Zhang, Zhaojiang Gong, Qianjin Liao, Guiyuan Li, Zhaoyang Zeng, Can Guo, Wei Xiong, He Huang

**Affiliations:** ^1^NHC Key Laboratory of Carcinogenesis and Hunan Key Laboratory of Cancer Metabolism, Hunan Cancer Hospital and the Affiliated Cancer Hospital of Xiangya School of Medicine, Central South University, Changsha, China; ^2^Department of Histology and Embryology, Xiangya School of Medicine, Central South University, Changsha, China; ^3^Department of Stomatology, Xiangya Hospital, Central South University, Changsha, China; ^4^Department of Oral and Maxillofacial Surgery, The Second Xiangya Hospital, Central South University, Changsha, China; ^5^Key Laboratory of Carcinogenesis and Cancer Invasion of the Chinese Ministry of Education, Cancer Research Institute, Central South University, Changsha, China

**Keywords:** nasopharyngeal carcinoma, long non-coding RNA, weighted gene co-expression network analysis, genomic instability, p53, MYC

## Abstract

Long non-coding RNAs (lncRNAs) widely regulate gene expression and play important roles in the pathogenesis of human diseases, including malignant tumors. However, the functions of most lncRNAs remain to be elucidated. In order to study and screen novel lncRNAs with important functions in the carcinogenesis of nasopharyngeal carcinoma (NPC), we constructed a lncRNA expression profile of 10 NPC tissues and 6 controls through a gene microarray. We identified 1,276 lncRNAs, of which most are unknown, with different expression levels in the healthy and NPC tissues. In order to shed light on the functions of these unknown lncRNAs, we first constructed a co-expression network of lncRNAs and mRNAs using bioinformatics and systematic biological approach. Moreover, mRNAs were clustered and enriched by their biological functions, and those lncRNAs have similar expression trends with mRNAs were defined as functional molecules with potential biological significance. The module may help identify key lncRNAs in the carcinogenesis of NPC and provide clues for in-depth study of their functions and associated signaling pathways. We suggest the newly identified lncRNAs may have clinic value as biomarkers and therapeutic targets for NPC diagnosis and treatment.

## Introduction

Nasopharyngeal carcinoma (NPC) is a kind of malignant tumor that originates from the nasopharyngeal epithelial cells (1). It is a common head and neck malignancy in southeast Asia and south China (2). However, NPC differs markedly from other head and neck malignancies in terms of epidemiology, etiology, pathological characteristics, and treatment strategies. NPC exhibits distinctive ethnic and regional distribution characteristics. In most parts of the world, the incidence of NPC is very low (1 in 100,000 people) but high in southeast Asia, north Africa, and other regions; in particular, the Guangdong province of south China has an incidence of NPC up to 30 in 100,000 people (3). NPC carcinogenesis is caused by pathogenic environmental factors and genetic factors (4). Environmental factors including exposure to carcinogenic chemicals (such as nitrosamines in food) and Epstein-Barr virus infection (5–12). Genetic factors, or genetic susceptibility, also plays an important role in the pathogenesis of NPC (13–15). Most cases of NPC are low-grade differentiated squamous cell carcinomas, which are relatively sensitive to radiation (16–19). Radiotherapy achieves good curative effects for patients with an early-stage of NPC, whose five-year survival rate exceeds 90%. However, due to the primary site of NPC is not readily visible, early symptoms of NPC are not evident, which makes it easy to be overlooked or misdiagnosed clinically. In addition, most NPC patients have a high degree of malignancy and a strong tendency to metastasis (20–24). Therefore, most patients with NPC occur metastases at the time of diagnosis and the effects of radiotherapy lose their in-time efficacy. Recurrence and metastasis are the main causes of treatment failure in NPC.

Many genes have been reported to be dysregulated and multiple signal transduction pathways function abnormally during NPC carcinogenesis (25–28). However, the mechanism of NPC development has not been fully elucidated. An increasing number of studies showed that, in addition to protein-coding genes, non-coding RNAs also play important roles in the development of malignant tumors. Most non-coding RNAs are longer than 200 nt and have been classified as long non-coding RNAs (lncRNAs) (29–31). At present, more than 90,000 lncRNA genes, encoding more than 140,000 transcripts, have been identified in the human genome (32), far more than the number of protein-coding genes. LncRNAs regulate gene expression at multiple levels, such as the epigenetic, transcriptional, and post-transcriptional levels, with important effects on biological functions (33–37). So far more than 100 lncRNAs have been found associated with NPC (38–42), however, these lncRNAs were just the tip of the iceberg. The roles and mechanisms of most lncRNAs in the development of NPC remain obscure. Therefore, a whole transcriptomic expression profile of lncRNA will enable effective screening and identification of important lncRNAs associated with NPC. Furthermore, it will enable us to explore the key driving factors during transformation of inflammation to carcinoma.

The identification of functinal lncRNAs remains difficult as many lncRNAs are undiscovered. However, regulation of protein-coding genes and non-coding RNAs is governed by certain rules (43). Genes with similar expression patterns may have common characteristics, such as may be regulated by common factors or participated in the same signaling pathway. Therefore, after we obtained lncRNA and mRNA expression profiles for NPC and the control tissues, we constructed the lncRNA–mRNA co-expression network based on their expression patterns. We defined functional modules by clustering and enriching mRNAs based on their biological functions and by determining lncRNAs with similar expression trends.

Another important cluster of RNA, the microRNAs (miRNAs), regulates gene expression in the transcriptome (44–46). miRNAs bind to other RNA molecules to induce the degradation of target RNA, thereby altering gene expression. LncRNAs and mRNAs that share the same miRNA binding site reciprocally regulate each other by competing for miRNAs (45). Although we did not use a gene chip with miRNA probes in this study, we performed miRNA target gene enrichment analysis through gene set enrichment analysis (GSEA) (47), to find RNAs share the same miRNA recognition site to build a competitive endogenous RNA (ceRNA) (48) co-expression network. We hypothesize that these functional modules may reveal lncRNAs with potential biological significance, which may provide clues for screening candidate lncRNAs and may aid the exploration of possible mechanisms of NPC.

## Materials and Methods

### Clinical Samples

We selected 6 non-cancerous nasopharyngeal epithelium (NPE) tissues and 10 NPC tissues for construction of lncRNA and mRNA expression profiles. And then we collected another 10 NPE and 26 NPC tissue samples for validation of lncRNA expression. All NPC tissues were from newly diagnosed patients who did not undergo treatment. The tissue specimens were stored in liquid nitrogen. This study was authorized by the Ethics Committee of the Central South University. All patients provided written informed consent.

### RNA Extraction, cDNA Synthesis, and Labeling

We minced the tissues (50 mg–100 mg) in liquid nitrogen and extracted total RNA using the TRIzol^®^ reagent according to the manufacturer’s instructions. We quantified RNA using a NanoDrop™ ND-2000 (Thermo Scientific) and assessed its integrity with an Agilent 2100 Bioanalyzer (Agilent Technologies). We reverse-transcribed double-stranded cDNA from RNA and synthesized a complementary RNA labeled with cyanine-3-CTP using the kit provided by Agilent.

### Chip Selection, Hybridization, and Image Acquisition

We profiled lncRNA and mRNA expression with the Agilent 4×180K lncRNA Array, which contains all known lncRNAs and mRNAs from multiple databases, such as NCBI RefSeq, UCSC, and Ensembl. The labeled complementary RNA was hybridized to the chip, then scanned with an Agilent Scanner G2505C (Agilent Technologies) after elution. Raw expression data were extracted from the images using the Agilent Feature Extraction software (version 10.7.1.1, Agilent Technologies). We standardized the data using GeneSpring software (version 13.1, Agilent Technologies) to obtain lncRNA and mRNA expression values from each sample for subsequent data analysis. The raw data for all the 16 tissues used in this project have been uploaded to the Gene Expression Omnibus (GEO) database (Accession number: GSE61218).

### Differential Expression Analysis

We filtered data to reduce the “background noise” for subsequent analysis. We retained lncRNAs or mRNAs that were detectable in at least one of the two sample groups (NPC or NPE). The filtered data were analyzed with the Significant Analysis of Microarray (SAM) software (49); standard parameters (fold change ≥ 1.5 and the false discovery rate q value ≤ 0.05) were used to identify significantly and differentially expressed transcripts. The differentially expressed lncRNAs and mRNAs were displayed in heatmap created using the Genesis software to visualize their expression patterns in the two groups of the samples.

### Cell Culture

NPC cell CNE2 was cultured in RPMI-1640 (Life Technologies, Grand Island, NY, USA) with 10% fetal bovine serum (FBS; Life Technologies) and 1% penicillin/streptomycin (Life Technologies), at 37 °C in a humid incubator with 5% CO2.

### Cell Transfection, RNA Extraction and qPCR

Cells were seeded in a 6-well plate and cultured overnight, the next day, cells were transfected with 50 nM siRNA and 5 μL Hiperfect (Qiagen). After 48 hours, total RNA was extracted with TRIzol (Invitrogen, CA, USA), 1 μg RNAs were reverse transcribed into cDNA using HiScript II Q RT SuperMix (Vazyme, Nanjing, China), and qPCR was performed using 2×SYBR Green qPCR Master Mix (Bimake, Changsha, China). Sequences of primers and siRNAs were shown in **Supplementary Table 1**.

### Wound Healing Assay

Cells were seeded in a 6-well plate and transfected with siRNAs or nc when cell density reached 50%. When the cells reached 100% confluency, the plate was scratched using a 10 µL pipette tip, making each gap as uniform as possible. The cells were cultured with 2% FBS and hydroxyurea (inhibit cell growth, 40 µg/mL). The gap was imaged at different time points using an inverted microscope (IX51, Olympus, Japan).

### Transwell Assay

Chambers (8-mm pores, Corning, NY, USA) were placed in a 24-well plate. Matrigel (BD Biosciences, NJ, USA) was diluted 1:9 with RPMI-1640 and 20 µL diluted Matrigel was added to the chambers following by incubating at 37 °C for 2h. Transfected cells were digested, and diluted to a density of 20,000 cells per 200 µL and then added to upper chamber and 800 µL 20% FBS was added to the bottom of 24-well plate. After incubation for 24-48 hours, the cells were fixed with 4% formaldehyde, stained with 0.1% crystal violet, and cells inside the chamber were wiped off, cells outside chamber were imaged using an inverted microscope (IX51, Olympus, Japan).

### CCK8

Cells were seeded in a 6-well plate and transfected with siRNAs or nc when cell density reached 50%. After 24 hours, the cells were digested and diluted to 800 per 200 µL, then cells were seeded in 96-well plates. Each group was set with 5 parallel wells. Then CCK8 (Hanbio, shanghai, China) was added and incubated for 2 hours, absorbance was measured at 450nm.

### Weighted Gene Co-Expression Network Analysis

LncRNAs and mRNAs share similar expression trends in the NPC and controls were calculated using the weighted gene co-expression network analysis (WGCNA) algorithm (50). We used those expression trends to construct the lncRNA–mRNA co-expression networks, which were visualized using the Cytoscape software.

### GSEA

GSEA is a computational method that determines whether *a priori* defined set of genes shows statistically significant, concordant differences between the two biological states, e.g. normal vs disease (47). We used GSEA to analyze the gene sets in which the differentially expressed molecules were enriched. Using a combination of the GSEA results and the lncRNA–mRNA co-expression network, we defined the lncRNA–mRNA modules that may share the characteristics of the enriched gene sets.

### Ingenuity^®^ Pathway Analysis

The Ingenuity^®^ Pathway Analysis (IPA^®^; http://www.ingenuity.com) is an integrated bioinformatic analysis software based on the Ingenuity Knowledge Base and the cloud computing provided by Qiagen (51). We imported data of our lncRNA and mRNA expression profile to obtain several potential core transcriptional regulatory factors. We then integrated those findings with the lncRNA co-expression network to construct a core transcription factor-driven lncRNA–mRNA co-expression module.

## Results

### Differentially Expressed lncRNAs and mRNAs in NPC

**Figure 1:**
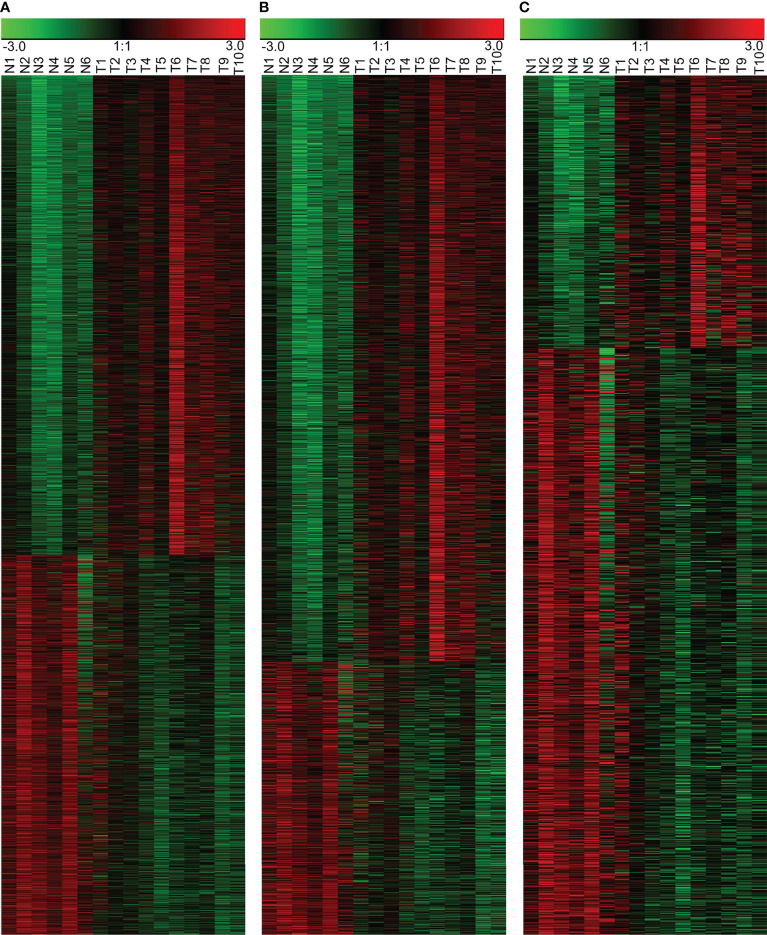
Differentially expressed RNAs in the NPC and the control NPE samples. **(A)** Heatmap of all differentially expressed RNAs, including lncRNAs and mRNAs. **(B)** Differentially expressed mRNAs. **(C)** Differentially expressed lncRNAs. N: normal nasopharyngeal epithelium; T: nasopharyngeal carcinoma.

We successfully profiled lncRNA and mRNA expression in 10 NPC tissues and 6 controls by a gene array. By filtering and analyzing data, we found 3,734 differentially expressed molecules, of which 1,276 were lncRNAs (405 up-regulated and 871 down-regulated in NPC) and 2,458 mRNAs (1,677 up-regulated and 781 down-regulated in NPC), as shown in **Supplementary Table 2**. A heatmap displaying the differentially expressed RNAs has been shown in **Figure 1**.

### Differentially Expressed lncRNAs Were Validated in NPC Tissues

**Figure 2:**
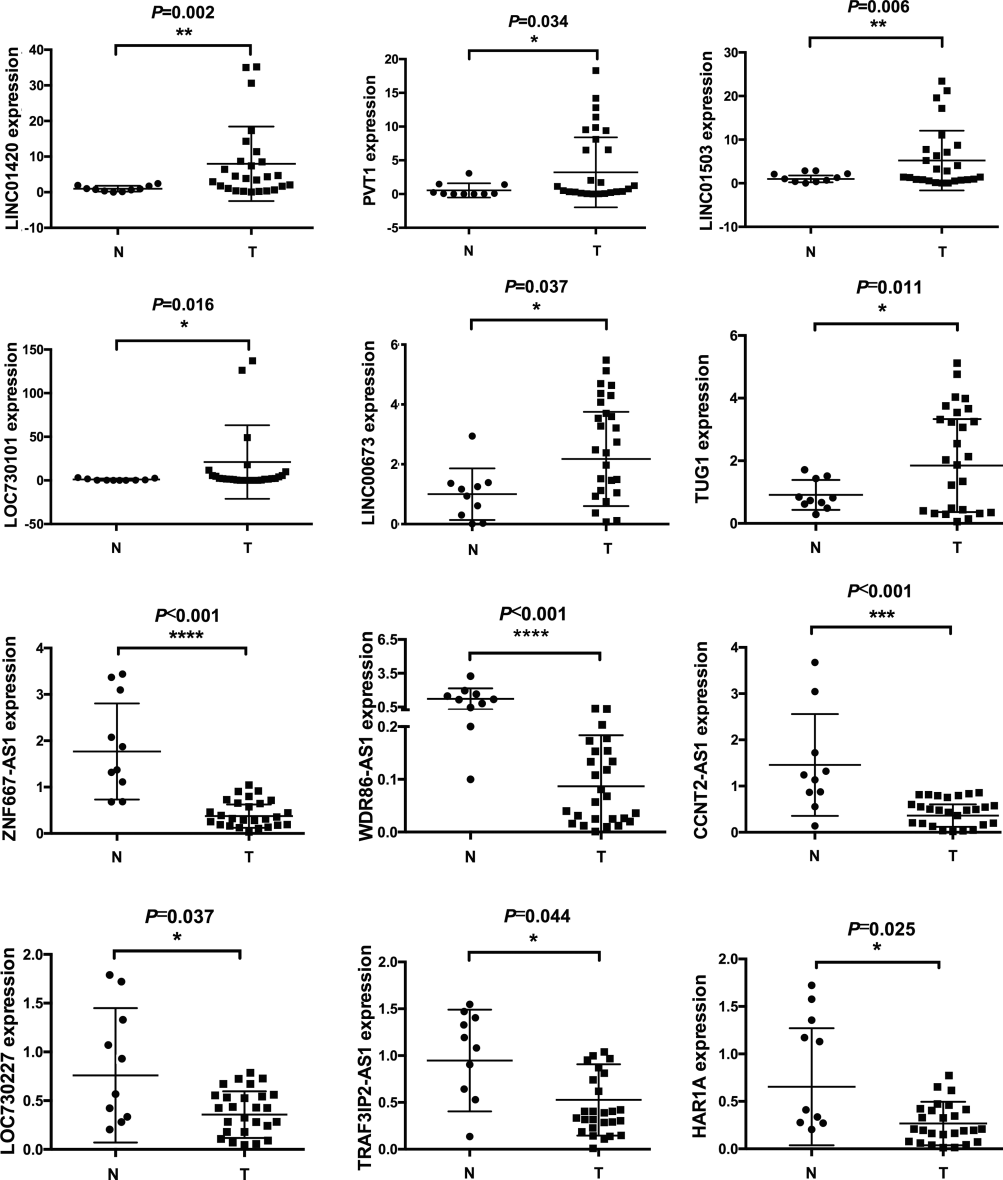
Differentially expressed lncRNAs were validated by qPCR. 10 normal and 26 cancerous tissues were used to detect expression levels of lncRNAs. Six upregulated and six downregulated lncRNAs were validated. * means p<05, ** means p<0.01, *** means p<0.001, **** means p<0.0001.

In order to verify the reliability of our gene array, we collected another 26 NPC tissues and 10 normal controls for qPCR detection. We randomly selected 6 lncRNAs from down-regulated or up-regulated modules, respectively. The results showed that LINC01420, PVT1, LINC01503, LOC730101, LINC00673, TUG1 was upregulated in NPC tissues, while ZNF667-AS1, WDR86-AS1, CCNT2-AS1, LOC730227, TRAF3IP2-AS1, HAR1A was downregulated in NPC tissues, which were consistent with gene array data (**Figure 2**).

### Biological Function of Differentially Expressed lncRNAs

**Figure 3:**
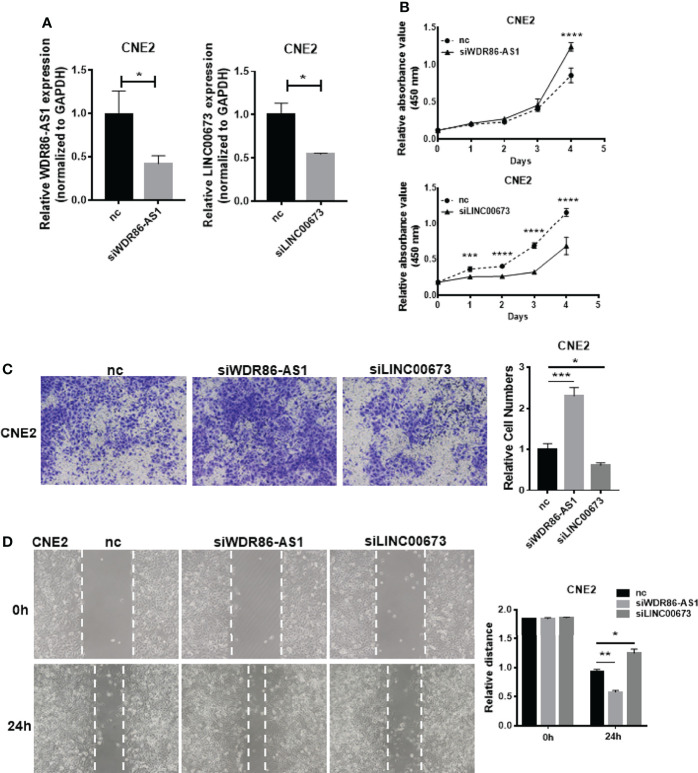
Biological function of differentially expressed lncRNAs. **(A)** The efficiency of knock down was detected by qPCR. **(B)** CCK8 was used to measure the proliferation ability of lncRNAs. **(C)** Transwell assays were performed to detect the invasion potential of lncRNAs after knock down. **(D)** Wound healing assays were employed to assess the migration rate. * means p<0.05, ** means p<0.01, *** means p<0.001, **** means p<0.0001.

To further prove that our differentially expressed lncRNAs have biological function. We selected WDR86-AS1 from down-regulated module and LINC00673 from up-regulated module for phenotype verification, which have not been reported in NPC. Firstly, the effect of knockdown using siRNAs was detected by qPCR (**Figure 3A**). CCK8 assays, transwell assays and wound healing assays demonstrated that siWDR86-AS1 promoted proliferation, invasion and migration ability of NPC cells, while siLINC00673 showed opposite effect (**Figures 3B-D**).

### Chromosome Co-Localization of Differentially Expressed lncRNAs and mRNAs

**Figure 4:**
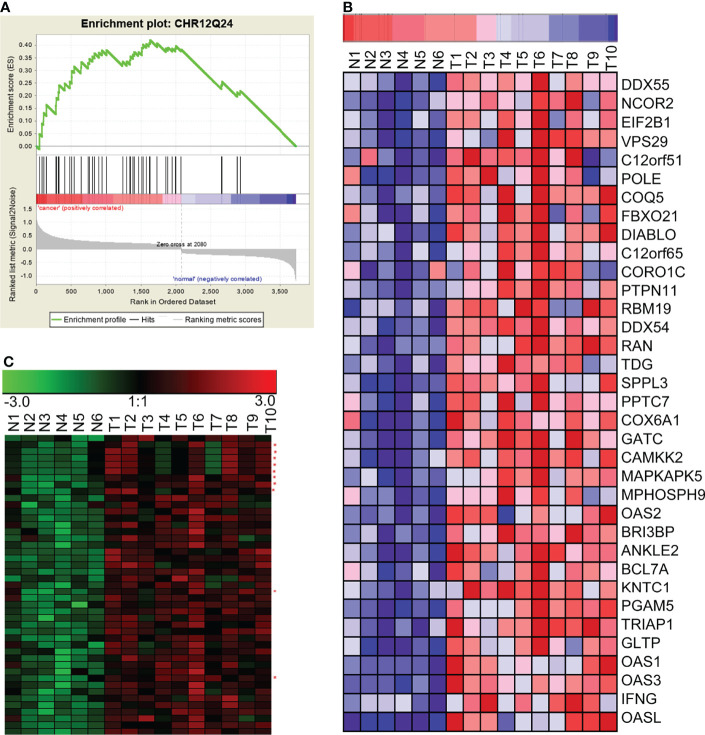
GSEA revealed the concurrent up-regulation of a branch of lncRNAs and mRNAs located on the chromosome 12q24 region. **(A)** GSEA showed that genes in the chromosome 12q24 region were significantly enriched in NPC. **(B)** mRNAs in the chromosome 12q24 region were significantly up-regulated in NPC. **(C)** lncRNAs and mRNAs in the chromosome 12q24 region were concurrently up-regulated (red asterisks beside the right of the heatmap indicate lncRNAs; the rest of the rows represent mRNAs). N, the nasopharyngeal epithelium; T, nasopharyngeal carcinoma.

Genomic instability, especially loss or amplification of chromosome fragments, is a special characteristic of NPC (1). The loss or amplification of certain chromosomal segments can alter gene expression in certain chromosomal region. Therefore, we investigated if the differentially expressed lncRNAs and mRNAs in NPC were enriched by a chromosomal localization. We analyzed differentially expressed genes with GSEA using gene set of chromosomal position (c1: positional gene sets). We found that there were significant enrichments in five chromosome segments. Of those, 12q24, 22p11, and 3q21 were significantly up-regulated together, suggesting that these chromosome segments may be amplified in NPC. However, the other two enriched segments, 3p21 and 11p15, were significantly down-regulated, suggesting that these chromosome segments may be absent in the NPC. The most significantly enriched chromosome is 12q24, and the expression patterns of mRNA and lncRNA in this chromosome segment have been shown in **Figure 4**, as an example of the chromosomal co-localization of the differentially expressed lncRNAs and mRNAs.

### Construction of a lncRNA and mRNA Co-Expression Network Using WGCNA

**Figure 5:**
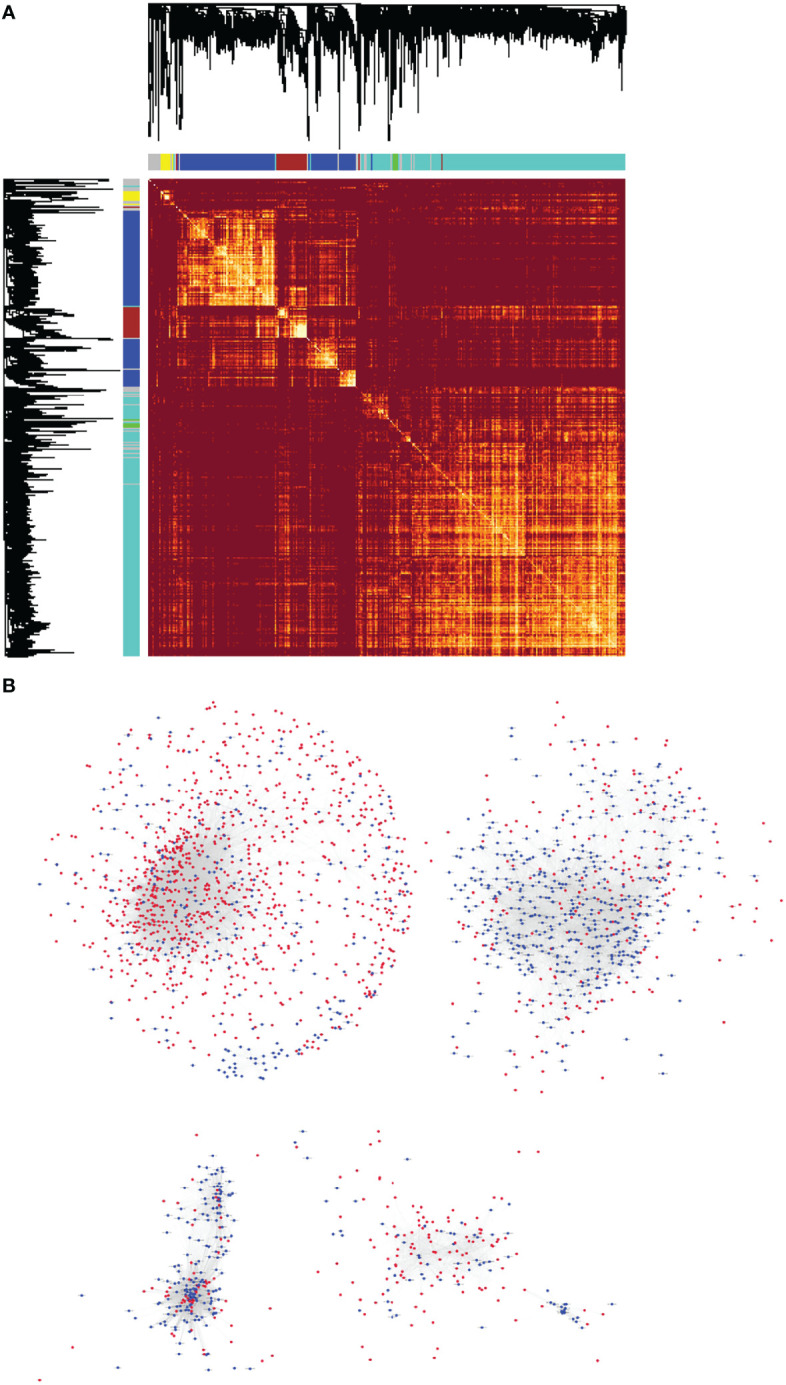
The lncRNA–mRNA co-expression network for NPC was constructed using WGCNA. **(A)** Heatmap of the topological overlap matrix of all the differentially expressed lncRNAs and mRNAs in NPC. The elements above and the left of the heatmap are hierarchical clustering trees. The different branches of the clustering tree represent different gene modules and have been displayed as different colored boxes. **(B)** Highly correlated, co-expressed lncRNAs and mRNAs with topological overlap greater than 0.09 were selected. They formed the basis of the lncRNA–mRNA co-expression network of NPC, which was illustrated using the Cytoscape software. There were 2,196 nodes (915 lncRNAs and 1,281 mRNAs) and 35,290 connections (or relationships) in the co-expression network.

At present, the functions of most lncRNAs in NPC remain unknown. However, we constructed the lncRNA–mRNA co-expression network to establish relationship between functionally annotated mRNAs and novel lncRNAs with unknown biological functions. We used the WGCNA algorithm to calculate the topological overlap between 3,734 differentially expressed RNAs and classify them according to their expression patterns, then we constructed a hierarchical clustering tree (**Figure 5A**, upper left). The branches of the clustering tree contained genes with similar expression patterns and represented a different gene module. We next constructed a correlation coefficient matrix of the differentially expressed RNAs (1,276 lncRNAs and 2,458 mRNAs, which formed a 3,734×3,734 matrix). The matrix has been represented as a heatmap (**Figure 5A**). Finally, we constructed the lncRNA–mRNA co-expression network for molecules with topological overlap greater than 0.09 (**Figure 5B**). This network included a total of 2,196 nodes (915 lncRNAs and 1,281 mRNAs) and 35,290 connections (relationships). The remaining 361 lncRNAs and 1,177 mRNAs did not exceed the threshold (0.09) for a co-expression relationship.

### ceRNA Modules Enriched by Common miRNA Binding Sites

**Figure 6:**
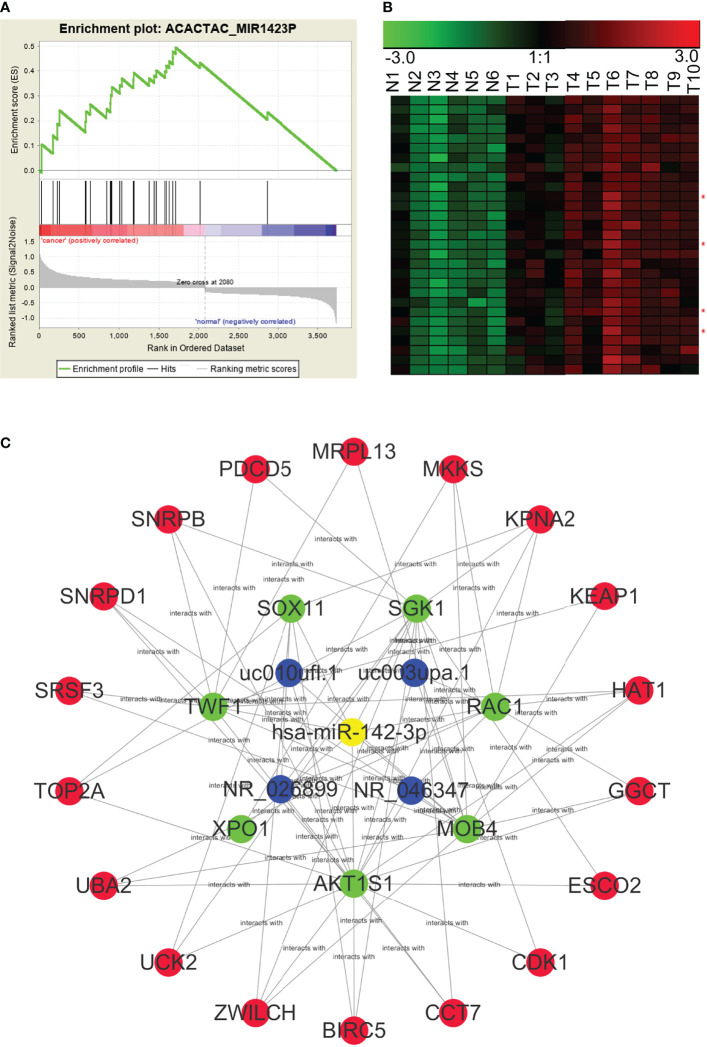
A potential NPC lncRNA–mRNA ceRNA module based on the competition for miR-142-3p. **(A)** GSEA predicted that miR-142-3p target genes were significantly enriched among the RNAs that were differentially expressed in NPC. **(B)** Expression profiles of lncRNAs and mRNAs that may be targeted by miR142-3p in NPC (red asterisks indicate lncRNAs). N, the nasopharyngeal epithelium; T, nasopharyngeal carcinoma. **(C)** An NPC lncRNA–mRNA regulatory module based on the competition for miR142-3p, constructed through GSEA and WGCNA.

Using the WGCNA algorithm, we determined the full lncRNA–mRNA co-expression network for NPC (**Figure 3B**). However, the biological significance of the lncRNA–mRNA modules was unclear. Using GSEA, we identified lncRNAs and mRNAs that shared miRNA recognition sites. We found that targets for miR-142-3p, miR506, and miR-17 family (including miR-17-5p, miR-20a, miR-106a, miR-106b, miR-20b, and miR-519d) were the most significantly enriched in the setting of NPC. **Figure 6** shows the lncRNA/miR-142-3p/mRNA ceRNA module as an example.

### Construction of lncRNA–mRNA Co-Expression Modules Based on Signaling Pathway

**Figure 7:**
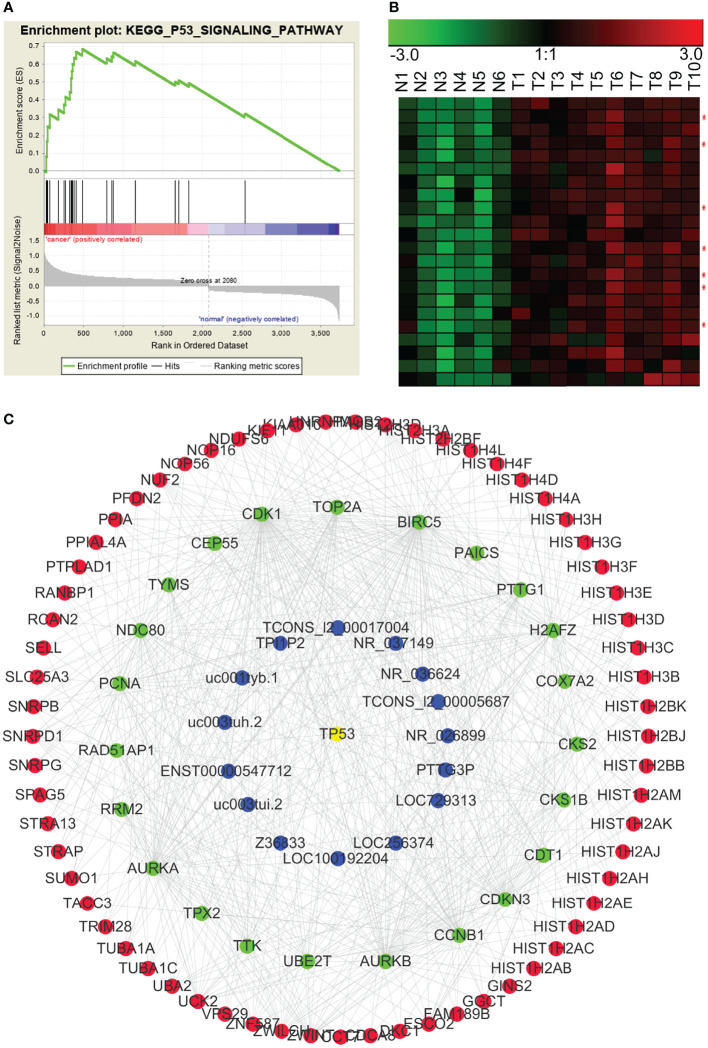
p53 pathway-related lncRNA-mRNA co-expression modules in NPC. **(A)** GSEA revealed that involvement in the p53 signal pathway was significantly enriched in the differentially expressed RNAs in NPC. **(B)** Many genes in the p53 pathway were significantly up-regulated in NPC (red asterisks indicate lncRNAs). N: the control nasopharyngeal epithelium, T, nasopharyngeal carcinoma. **(C)** We used GSEA and WGCNA to construct the lncRNA–mRNA co-expression module related to the p53 signaling pathway.

In addition to finding lncRNA–mRNA co-expression modules based on the chromosomal co-localization and the ceRNAs, we enriched modules based on signaling pathways. Using GSEA, we analyzed gene sets that contained all the pathways from the Kyoto Encyclopedia of Genes and Genomes (KEGG) to enrich for the signaling pathways associated with the differentially expressed RNAs. We found that the p53 signaling pathway (KEGG_P53_SIGNALING_PATHWAY), the cell cycle regulatory pathway (KEGG_CELL_CYCLE), and the tumor-associated pathway (KEGG_PATHWAYS_IN_CANCER) were significantly enriched in NPC. We then used WGCNA to construct the lncRNA–mRNA co-expression modules based on the signaling pathways. In **Figure 7**, the p53 pathway has been used as an example of the lncRNA–mRNA co-expression module for an enriched pathway.

### Classification of lncRNA–mRNA Co-Expression Modules by Key Transcriptional Regulatory Factors

**Figure 8:**
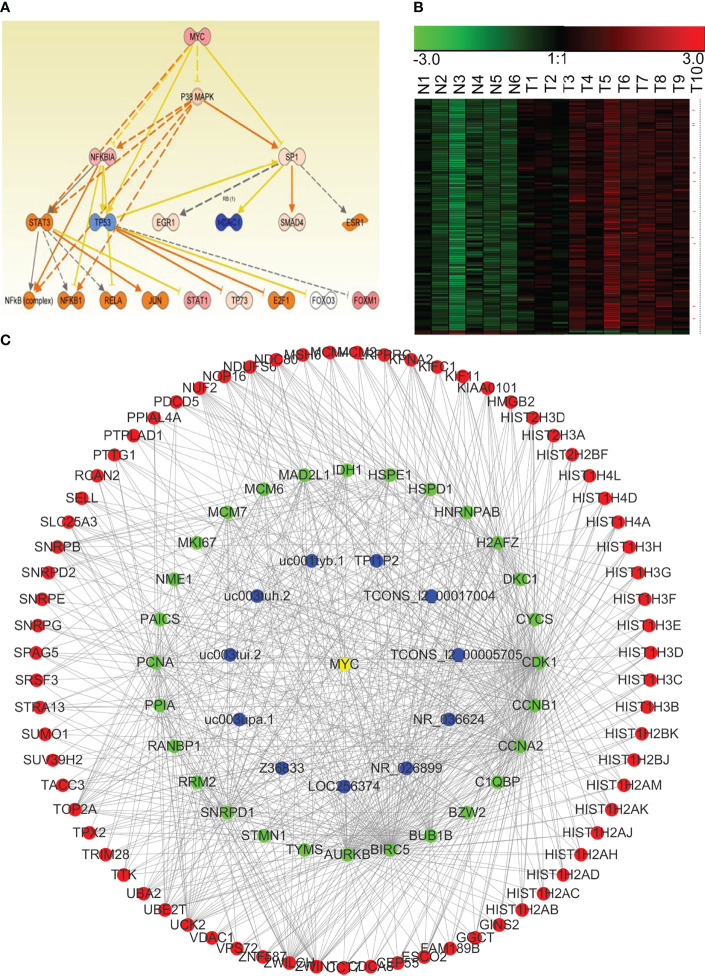
The MYC-driven lncRNA–mRNA co-expression network module in NPC. **(A)** The regulatory model for MYC and the other transcription factors involved in NPC (obtained using IPA). **(B)** Expression of the potential mRNAs and lncRNAs downstream of MYC in NPC (red asterisk indicates lncRNAs). N, the control nasopharyngeal epithelium; T, nasopharyngeal carcinoma. **(C)** We generated a MYC-driven lncRNA–mRNA co-expression network module for NPC by integrating the IPA and the WGCNA results.

Transcriptional regulatory factors drive the transcription of many genes, especially in the process of carcinogenesis. Therefore, assessment on co-regulation of lncRNA–mRNA co-expression network is important for the determination of regulatory mechanism of NPC. We used IPA to perform an integrated analysis of all differentially expressed lncRNAs and mRNAs, and found that β-estradiol, MYC, p53, E2F4, and ERBB2 were important upstream regulatory factors in the NPC transcriptome. We integrated the analyses from IPA and WGCNA, and constructed the lncRNA–mRNA co-expression modules that were driven by these core transcriptional regulatory factors. **Figure 8** shows the MYC-driven lncRNA–mRNA co-expression module as an example.

## Discussion

The study of lncRNAs is a hotspot of biomedical research due to the abundance of lncRNAs, their extensive participation in the regulation of other genes, and their roles in the development of a variety of human diseases (52–54). Research about lncRNAs is at the frontier of science as only a small portion of lncRNAs has been studied. The biological functions and molecular mechanisms of most lncRNAs remain unknown. The construction of lncRNA expression profiles and screening for differentially expressed lncRNAs are critical for the identification of disease-relevant lncRNAs and their pathogenic mechanisms. Given the generally low incidence of NPC globally, the small size of the nasopharyngeal cavity, and the limited availability of biopsy tissues, there have been few reports on lncRNA expression profiles in NPC (55–57). In this study, we constructed lncRNA and mRNA expression profiles using 10 NPC tissues and 6 normal controls, which were uploaded to the public database. These profiles will provide a basis for further screening and multi-center verification of lncRNAs that play an important role in the carcinogenesis of NPC.

We identified 1,275 lncRNAs and 2,485 mRNAs that were significantly and differently expressed in the NPC samples compared to controls. Among these differentially expressed lncRNAs, some have been previously reported associated with NPC; for example, AFAP1-AS1 (41), LOC284454 (42), PVT1 (16) and LINC01420 (40) have been reported upregulated in NPC tissues and promoted migration and invasion ability of NPC cells, these findings verified the reliability of our gene array data. However, most of the other lncRNAs have not been reported in the literature. Screening for important lncRNA molecules for subsequent research will not only expand our knowledge about pathogenesis of NPC, but also provide new annotations for functions of these lncRNAs.

Among these differentially expressed lncRNAs, some may be driving factors of nasopharyngeal epithelial carcinogenesis (58). In contrast, other differentially expressed lncRNAs may be simply associated with the carcinogenesis, which may be caused by a disordered transcriptional regulation during carcinogenesis (59). The ability to find true driving factors (lncRNAs) is the key to further success. Fortunately, we used gene chip technology to simultaneously obtain the mRNA expression profile while constructing the lncRNA expression profile of NPC, and most of the protein-coding genes were found to have functional annotations. This allowed us to cluster lncRNAs and mRNAs with similar expression patterns, which provided clues for the unknown functions of these lncRNAs.

WGCNA is a systematic biological algorithm to describe patterns of gene association between different samples (60). Compared with the traditional one-size-fits-all, hard-threshold algorithm, WGCNA sets a soft threshold and calculates the correlation coefficient weighting value, so that the connections between genes in the network obey scale-free networks. We found the sensible biological flexibility of the algorithm. We used the WGCNA algorithm to analyze the 1,275 differentially expressed lncRNAs and 2,485 differentially expressed mRNAs in NPC, and found the co-expression correlations between 915 lncRNAs and 1,281 mRNAs. We constructed the first lncRNA–mRNA co-expression network of NPC, which contained 35,290 correlations. The network provides important information for studying the functions of new lncRNAs that drive NPC carcinogenesis.

Genomic instability is one of the biological characteristics of malignant tumors. The frequency of point mutations in the NPC genome is the lowest while variation in the copy number occurs widely in the NPC genome (61–63). There are two copies of genes on the autosomes, both of which may be transcribed in normal cells. If a chromosome segment is lost or amplified during carcinogenesis, the change in the chromosome may result in down-regulation or up-regulation of genes on the chromosome. In this article, we used GSEA to cluster the chromosomal positions of the differentially expressed RNAs in NPC and found that genes in the chromosome regions 12q24, 22p11, and 3q21 were significantly up-regulated, suggesting that these chromosome segments may be amplified. There may be oncogenes, including oncogenic lncRNAs, in these segments. Genes in the chromosome segments 3p21 and 11p15 were significantly down-regulated. The down-regulation of the tumor suppressor genes, including lncRNAs, in these two chromosome segments may be an initiating factor for NPC. This hypothesis is consistent with previous studies of genomic instability in NPC (64–68).

One of the most important ways for lncRNAs to exert their biological functions is to act as sponges by competitively adsorbing miRNAs, thus indirectly regulating the expression of other mRNAs (69–71). The ceRNA hypothesis was first proposed by Professor Pandolfi in 2011. He posited that RNA molecules (mRNAs or lncRNAs) share miRNA response elements, or miRNA-binding sites, may compete with miRNA, thereby regulating each other’s expression and forming a large, complex ceRNA regulatory network in cells (48). The probes used in our gene chip were 60 mers that were not suitable for detecting miRNAs (~20 nt long). However, there are gene sets of miRNA target genes in the molecular signatures database (MSigDB) in GSEA. GSEA classifies genes by sharing an miRNA-binding site (47). Using GSEA, we successfully enriched a series of target genes that share binding sites for individual miRNAs. We combined those results with our WGCNA results to construct competitive endogenous lncRNA–mRNA co-expression modules. These modules provide important functional clues for future study of lncRNAs driving NPC. For example, miR-142-3p is an important tumor suppressor miRNA (72–74). Using GSEA and WGCNA, we suggest that a group of mRNAs and lncRNAs with similar expression trends in NPC may share a miR-142-3p-binding site. These newly identified lncRNAs may play an important role in the development of NPC by competing for miR-142-3p, thereby regulating some important mRNAs, such as *BIRC5*, *CDK1*, and *TOP2A*. Based on this finding, we propose further in-depth research using the co-expression networks to yield new discoveries about the mechanisms governing NPC.

We also performed a pathway enrichment analysis on differentially expressed genes in NPC using the KEGG. As expected, the p53 signaling pathway, cell cycle pathway, and pathways in cancer were most significantly enriched. The KEGG pathways in cancer refer to a large signaling pathway formed by the integration of multiple signaling pathways related to tumor development, including p53 and cell cycle pathways. Abnormal cell cycle regulation is a fundamental aspect of malignant tumors (75), and the *TP53* gene is one of the most important tumor suppressor genes. The p53 protein encoded by this gene is widely involved in the initiation and development of malignant tumors. The enrichment of these signal pathways in our analysis indicates that our results of gene chip are reliable. It also suggests that lncRNAs enriched in these signaling pathway modules, as indicated by GSEA and WGCNA, may be regulated by p53 or may participate in the regulation of the p53 signaling pathway (76). Similarly, we found potential core transcriptional regulatory factors that may drive the dysregulation of NPC transcriptome through IPA. Among these factors, MYC (77), p53, and E2F4 are important nuclear transcription factors that regulate expression levels of genes involved in cell cycle, apoptosis, metabolism, and other cell functions (78–81). For example, a series of important proteins, such as MAPK (82), NF-κB (83), and STAT3 (84), may be regulated by c-Myc (85); there are series of lncRNAs and mRNAs that are regulated by these proteins, which constitute complex lncRNA–mRNA co-expression network modules. The lncRNAs in these modules may also be important in NPC carcinogenesis.

Noteworthy, the core regulatory factors β-estradiol and ERBB2 were enriched according to the IPA. β-estradiol is an estrogen that activates the estrogen receptor and regulates the expression of downstream genes (86–89). The protein encoded by ERBB2 is the well-known oncoprotein HER-2 (90). β-estradiol and HER-2 have been known to be the main driving factors of breast cancer. The expression of estrogen receptor and HER-2 remain as the main criteria for the clinicopathological classification of breast cancer (86–90). However, their roles in NPC have not been well-studied. Recent reports suggest that the estrogen receptor may be a tumor suppressor gene, or a protective factor, in tumors such as gastric cancer (91). Given the lower estrogen levels in men, a role for the estrogen receptor as a tumor suppressor may be a contributing factor to the higher incidence of NPC in men than women (92). Therefore, β-estradiol- and HER-2-driven downstream lncRNA–mRNA regulatory modules, especially the lncRNAs, constitute an exciting new area of research into the pathogenesis of NPC.

In conclusion, the role of lncRNAs in the pathogenesis of NPC remains to be elucidated, and a large number of functional lncRNAs have not been thoroughly studied. For the first time, we constructed a co-expression network of lncRNAs and mRNAs in the transcriptome of NPC using bioinformatics and systematic biological methods. Through functional clustering and the enrichment of differentially expressed mRNAs in NPC, we identified functional modules with potential biological significance for carcinogenesis of NPC. These modules will aid in the discovery of key lncRNAs in NPC and will provide clues for the mechanistic study of novel lncRNAs.

## Data Availability Statement

The datasets presented in this study can be found in online repositories. The names of the repository/repositories and accession number(s) can be found in the article/**Supplementary Material**.

## Ethics Statement

The tissue specimens were stored in liquid nitrogen. This study was authorized by the Ethics Committee of the Central South University. All patients provided written informed consent.

## Author Contributions

CF contributed to drafting and editing the manuscript. WX and HH designed, revised, and finalized the manuscript. FX, YT, and PL participated in the drafting. KZ, YM, and YW participated in the revision. SZ, ZG, QL, GL and ZZ, CG contributed to the literature search. All authors contributed toward data analysis, drafting, and revising and agreed to submit. All authors contributed to the article and approved the submitted version.

## Funding

This work has been supported by the National Natural Science Foundation of China (81872278, 81803025 and 81972776), the Natural Science Foundation of Hunan Province (2018JJ3815, 2018SK21210, 2018SK21211), and open sharing fund for the large-scale instruments and equipments of Central South University(CSUZC202235).

## Conflict of Interest

The authors declare that the research was conducted in the absence of any commercial or financial relationships that could be construed as a potential conflict of interest.

## Publisher’s Note

All claims expressed in this article are solely those of the authors and do not necessarily represent those of their affiliated organizations, or those of the publisher, the editors and the reviewers. Any product that may be evaluated in this article, or claim that may be made by its manufacturer, is not guaranteed or endorsed by the publisher.

## Supplementary Material

Supplementary Table 2 Upregulated and downregulated mRNA or lncRNAs.

Supplementary Table 3 Clinical information of NPC patients.

## Abbreviations

ceRNA, competitive endogenous RNA; GEO, Gene Expression Omnibus; GSEA, gene set enrichment analysis; IPA, Ingenuity Pathway Analysis; KEGG, Kyoto Encyclopedia of Genes and Genomes; lncRNA, long non-coding RNA; miRNA, microRNA; MSigDB, molecular signatures database; NPC, nasopharyngeal carcinoma; NPE, nasopharyngeal epithelium; SAM, Significant Analysis of Microarray; WGCNA, weighted gene co-expression network analysis.
