# Supplementary material for: Construction of a lncRNA–mRNA Co-Expression Network for Nasopharyngeal Carcinoma
Source: Front Oncol. 2022 Jul 7;12:809760. doi: 10.3389/fonc.2022.809760 (PMC9302896; doi:10.3389/fonc.2022.809760)
Supplement: Supplementary file 1 [file Table_1.docx]

**Supplementary table 1: primers, siRNAs**

| **primers for real-time PCR** | |
| --- | --- |
| LINC01420-F | 5'-CACTCTACCCTCCGCACC-3' |
| LINC01420-R | 5'-AGGAAGTGAAATCGTGCTGA-3' |
| PVT1-F | 5'-TGGCTGAGAGGGTTGAGATC-3' |
| PVT1-R | 5'-GCTGTATGTGCCAAGGTCAC-3' |
| LINC01503-F | 5'-TCCCAAAGTGCAGGGATTAC-3' |
| LINC01503-R | 5'-GGGGGACTGATGATGAACAC-3' |
| LOC730101-F | 5'-ACCAAGAGGGTTGACGTTTG-3' |
| LOC730101-R | 5'-CTTCTCCTCAGTGGCTTTGG-3' |
| LINC00673-F | 5'-TTCTCCTGTAACGTGTGGCC-3' |
| LINC00673-R | 5'-CTGGTGGGAATGTGGATCAGT-3' |
| TUG1-F | 5'-CACAAGGCTGCACCAGATT-3' |
| TUG1-R | 5'-CTGGGCTTCTGCACAGTTTT-3' |
| ZNF667-AS1-F | 5'-AGTGTCCGCCATAAAGTCGT-3' |
| ZNF667-AS1-R | 5'-AGAGATCGTAGCAGGGTCCA-3' |
| WDR86-AS1-F | 5'-ACACACCGCTGACCTTACCT-3' |
| WDR86-AS1-R | 5'-CTTCCCGTTTTCTCTCGACT-3' |
| CCNT2-AS1-F | 5'-ATGCCACTCATGGAGCAACT-3' |
| CCNT2-AS1-R | 5'-TCCCTTGGCTTGTAGGTTTG-3' |
| LOC730227-F | 5'-CATTTCCAGCTCAGAAGCAAC-3' |
| LOC730227-R | 5'-TGTCCCGATCATTACCCAAC-3' |
| TRAF3IP2-AS1-F | 5'-CCGCTTCTGTGGTTTCTGTC-3' |
| TRAF3IP2-AS1-R | 5'-GGCTTTTCCACTTCAATCCA-3' |
| HAR1A-F | 5'-CAACCCGCAGACCATGTAA-3' |
| HAR1A-R | 5'-TGACTGGGCAGCTCTTCTCT-3' |
| β-actin-F | 5'-TCACCAACTGGGACGACATG-3' |
| β-actin-R | 5'-GTCACCGGAGTCCATCACGAT-3' |
| **siRNAs** |  |
| si-LINC00673 | GGTGGAATCAGAGGTTTCT |
| si-WDR86-AS1 | AGATCTAACAGGAGTACAA |
