# Supplementary material for: Construction of a lncRNA–mRNA Co-Expression Network for Nasopharyngeal Carcinoma
Source: Front Oncol. 2022 Jul 7;12:809760. doi: 10.3389/fonc.2022.809760 (PMC9302896; doi:10.3389/fonc.2022.809760)
Supplement: Supplementary Table 3 — Clinical information of NPC patients. [file Table_3.docx]

| Number | Gender | Age | Pathological  type | T stage | N stage | M stage | Clinical stage |
| --- | --- | --- | --- | --- | --- | --- | --- |
| 1 | Male | 70 | Undifferentiated | 2 | 2 | 1 | IV |
| 2 | Female | 35 | Undifferentiated | 3 | 2 | 0 | III |
| 3 | Male | 50 | Undifferentiated | 2 | 2 | 0 | III |
| 4 | Male | 50 | Undifferentiated | 2 | 3 | 0 | IV |
| 5 | Female | 46 | Undifferentiated | 2 | 2 | 0 | III |
| 6 | Male | 48 | Low differentiated | 2 | 3 | 0 | IV |
| 7 | Male | 47 | Undifferentiated | 4 | 2 | 0 | IV |
| 8 | Male | 40 | Mixed | 3 | 2 | 0 | III |
| 9 | Male | 59 | Undifferentiated | 2 | 3 | 0 | IV |
| 10 | Female | 58 | Low differentiated | 2 | 2 | 0 | III |
| 11 | Male | 43 | Undifferentiated | 2 | 2 | 1 | IV |
| 12 | Male | 48 | Low differentiated | 2 | 2 | 0 | III |
| 13 | Male | 49 | Undifferentiated | 3 | 2 | 0 | III |
| 14 | Male | 46 | Undifferentiated | 1 | 2 | 0 | III |
| 15 | Male | 47 | Low differentiated | 2 | 2 | 0 | III |
| 16 | Male | 48 | Mixed | 2 | 2 | 0 | III |
| 17 | Male | 51 | Undifferentiated | 2 | 3 | 0 | IV |
| 18 | Female | 23 | Low differentiated | 4 | 2 | 0 | IV |
| 19 | Male | 57 | Undifferentiated | 1 | 3 | 0 | IV |
| 20 | Male | 39 | Undifferentiated | 4 | 2 | 0 | IV |
| 21 | Male | 33 | Undifferentiated | 4 | 2 | 0 | IV |
| 22 | Female | 64 | Low differentiated | 3 | 3 | 0 | IV |
| 23 | Female | 39 | Undifferentiated | 2 | 2 | 0 | III |
| 24 | Female | 33 | Low differentiated | 2 | 2 | 0 | III |
| 25 | Female | 44 | Undifferentiated | 1 | 3 | 0 | IV |
| 26 | Female | 38 | Mixed | 4 | 2 | 0 | IV |
